# Effects of substituting soybean meal with corn on immune function and gene expression of gut TLR4 pathway of growing goats

**DOI:** 10.7717/peerj.12910

**Published:** 2022-02-07

**Authors:** Yan Cheng, Chao Yang, Wenxun Chen, Qiongxian Yan, Zhiliang Tan, Zhixiong He

**Affiliations:** 1 CAS Key Laboratory for Agro-Ecological Processes in Subtropical Region, National Engineering Laboratory for Pollution Control and Waste Utilization in Livestock and Poultry Production, Hunan Provincial Key Laboratory of Animal Nutritional Physiology and Metabolic Process, Institute of Subtropical Agriculture, The Chinese Academy of Sciences, Changsha, Hunan, China; 2 University of Chinese Academy of Sciences, Beijing, China; 3 Hunan Co-Innovation Center of Animal Production Safety, CICAPS, Changsha, Hunan, China

**Keywords:** Protein, Immune function, TLR4 pathway, Gut, Growing goats

## Abstract

**Background:**

Protein malnutrition remains a severe problem in ruminant production and can increase susceptibility to infection, especially during the growth stage. This study aimed to explore substituting soybean meal with corn on activation of the TLR pathway and potential impact on immune response bias towards Type 1 or Type 2 using growing female goats as experimental animals.

**Methods:**

Twenty-four Xiangdong black goats (initial BW = 19.83 ± 0.53 kg, about 8 ± 0.3 months old) were selected and randomly divided into the corn-soybean meal basal diet group (CON, 10.77% protein) and replacing soybean meal with 100% of corn group (CRS, 5.52% protein). EDTA whole blood and serum samples were collected prior to slaughter for determinations of blood cell counts, anti-inflammatory cytokines and antibodies. The duodenum, jejunum, ileum and colon tissues were collected after formal trial to study the effect of CRS diet on the expression of TLR4 pathway.

**Results:**

Our results showed CRS diet did not induce a significant change in immune function, as evidenced by the observations that white blood cell (WBC), neutrophil (Neu), lymphocyte (Lym), monocyte (Mon), eosinophil (Eos), interleukin-4 (IL-4), IL-5, IL-13, immunoglobin G (IgG), IgA, and IgM levels in serum were similar between the two groups. RT-PCR results showed the expression of tumor necrosis factor-α (TNF-α) (*P* < 0.01) and interferon-β (IFN-β) (*P* < 0.01) were up-regulated in the colon of goats in the CRS group. No differences in the expression of myeloid differentiation factor 88 (MyD88) adaptor-like protein (TIRAP), IL-1 receptor–associated kinase 1 (IRAK1), TNF receptor related factor 6 (TRAF6), NF-kappa B (NF-κB), mitogen-activated protein kinase 1 (MAPK1) or activator protein-1 (AP-1) in the TLR4/MyD88 dependent pathway were observed between the two groups for any of the tested tissue. However, the expression of NF-κB activator (TANK) binding kinase 1 (TBK1) in TLR4/MyD88 independent pathway was up-regulated in the duodenum and colon (*P* < 0.01), and the expression of interferon regulatory factor-3 (IRF3) was up-regulated (*P* < 0.01) in colon.

**Conclusions:**

Our results suggested that the CRS diet failed to induce a significant change in innate immunity and adaptive immunity in growing goats. However, the up-regulated TBK1 and IRF3 in the colon from the CRS goats suggests that the CRS diet may induce the expression of Th1-type proinflammatory cytokines and inflammatory response through a TLR4-MyD88-independent pathway, and the colon may be the easiest targeted section in the intestinal tract.

## Introduction

Adequate protein in the diet is a vital source of essential amino acids that play an important role in the physiological process (Wu, 2009; de Vries-ten Have et al., 2020). Soybean meal is among the most common sources of protein in the diet of ruminants. Forage resources in arid or semi-arid regions are limited and feeding an adequate and biologically available protein to livestock might be difficult or even impossible (Firmenich et al., 2020). In ruminants, the excessive dietary nitrogen converted to ammonia in rumen and produced urea in the liver, which was excreted in the urine and resulted in water pollution, soil acidification, and other environmental problems (Hristov et al., 2011). Moreover, protein is the most expensive component of animal rations. A low protein diet is desirable for economic and environmental reasons in ruminant feeding. Due to efficient rumino-hepatic circulation of urea (Muscher et al., 2010), ruminants can recycle N more effectively than monogastric species to cope with a reduced protein and therefore diminished N intake as long as the energy supply is adequate (Firmenich et al., 2020). Williams, Verstegen & Tamminga (2001) reported that about 68% of the energy value of fermented carbohydrates was provided to the host as the form of volatile fatty acids. As the least expensive and an excellent carbon and energy source in diets (Jeong et al., 2021), carbohydrate has a protein-sparing effect in ruminants.

Several human and animal studies have shown that protein malnutrition triggers immune dysfunction, including altered innate and adaptive immune responses, impairment of immune system and increased the risk of infectious disease (Rytter et al., 2014; Vlasova et al., 2017; Miyazaki et al., 2018). The innate immune system consists of phagocytic cells (*e.g*., neutrophils, monocytes, macrophages, and dendritic cells), natural killer (NK) cells, and cells that release inflammatory mediators (basophils, mast cells, and eosinophils) (Carroll & Forsberg, 2007). Cellular indicators such as white blood cells, lymphocytes, neutrophils, monocytes, basophils and eosinophils were used to reflect the change of immune function in growing goats feed a substitution of soybean meal with corn diet in this study. The innate immune system is the first line of defense against invading pathogens by either direct mechanisms such as phagocytosis and cytokine production or indirectly by activating adaptive immunity (Mian, Paradis & Schiffrin, 2014). Adaptive immune system, which consists of cell mediated (T cell--mediated) and humoral immunity (B cell--mediated), is characterized by the production of inflammation-related cytokines and antibodies respectively (Wolf & Ley, 2019).

The cytokines secreted by T-helper 1 (Th1) cells, including tumor necrosis factor-alpha (TNF-α), interferon-gamma (IFN-γ), interleukin-1 beta (IL-1β), and IL-2, are involved in cell-mediated immunity while Th2 cytokines (IL-4, IL-5, and IL-13) have shown anti-inflammatory effect and promote humoral immunity (Ewer et al., 2021; Schulze-Koops & Kalden, 2001). The studies in dam and mice showed that interferon-beta (IFN-β) could inhibit the release of IFN-γ and also has anti-inflammatory and pro-inflammatory effects (Boivin et al., 2015; Bolivar et al., 2018). It has been reported that the cells in neonatal goats displayed a more robust Th-1 response to various TLR agonists than adult cells (Tourais-Esteves et al., 2008). In a general statement, there may be a specific immune response bias modulation in a susceptible environment. B cells can mediate different biological effector functions by switching from immunoglobin M (IgM) to IgG, IgA or IgE expression ( Brightbill et al., 2010). It has been reported that IFN-γ can promote the secretion of IgG2a and IgG3 antibodies, while Th2 cytokines, such as IL-4, IL-5, and IL-13, arouse IgG1 antibody production by B cells ( Kovacs-Nolan et al., 2009). The release of anti-inflammatory cytokines and specific antibodies by the adaptive immune system, are important in protecting from infections (Nakayama et al., 2017). Thus, cytokines and antibody levels were used as the targets to evaluate the immune activities of protein restricted goats.

The intestine also serves as the largest compartment of the immune system and contains the largest number of immune cells of any tissue in the body (Vighi et al., 2008; Mowat & Agace, 2014). Toll-like receptors (TLRs) are a family of at least 10 proteins that functions as pattern recognition receptors (PRRs) and play an essential role in animal immunity (Medzhitov, 2001; Vandana et al., 2019). TLR4 is an important member of the TLR family in the gut. It has been recognized as a receptor for lipopolysaccharide (LPS), a component of the outer membrane of Gram-negative bacteria that can activate TLR4/ myeloid differentiation factor 88 (MyD88)-dependent and TLR4/MyD88-independent signaling pathways ( Brown et al., 2011), resulting in production of pro-inflammatory cytokines and strong inflammatory response (Akira, Takeda & Kaisho, 2001; Ren et al., 2019). Since malnutrition, in particular the protein–energy malnutrition is closely related to inflammation (Sauerwein et al., 1997). We hypothesized that protein deficiency can cause inflammatory disorder in the gut and there may be a Th1-type response bias in goats exposed to a highly infectious environment due to protein malnutrition. The present study may help shed new light on applied nutrition approaches to improve malnourished goat performance and health during the critical life-stage transition (growth stage).

In addition, previous studies have suggested malnutrition presents a greater risk in female goats during growth, pregnancy and lactation periods (Heersink et al., 2010; Castel, Shahar & Harman-Boehm, 2006). In the present study, we focus on reducing dietary protein under isoenergetic conditions, and an extremely low protein diet was formulated by replacing soybean meal with corn. The objective of this study was to evaluate the effect of substituting soybean meal with corn on activation of the TLR pathway and potential impact on immune response bias towards Type 1 or Type 2 (immune cellular indicators, anti-inflammatory cytokines and antibody levels) using growing female goats as experimental animals. Through the model for ruminant nutrition, gaining a deeper understanding between protein nutrition and immune function.

## Materials and Methods

All procedures were approved by the Institutional Animal Care and the Use Committee of the Institute of Subtropical Agriculture, Chinese Academy of Sciences, Changsha, China.

### Animals and experimental design

Twenty-four healthy female goats were purchased from a commercial goat farm (Pingxiang, Jiangxi province, China). Health scores (nasal score, fecal score, attitude score) were collected as previously described (Cheng et al., 2021). Cough scores were categorized as 0: never cough; 1: single cough; 2: repeated cough or occasional spontaneous cough; 3: repeated spontaneous cough. Each nasal score, fecal score, attitude score and cough score received a score 0 was considered as healthy.

All goats were eartagged and moved to straw-bedded individual cages (1.2 × 0.9 m) at the experimental station of the institute of Subtropical Agriculture, Chinese Academy of Sciences (Hunan province, China) during the period from October 27th, 2018 to January 21th, 2019 (ISA-2019-0115). The trial consisted of three experimental periods. Preparation period lasted for one month (October 27th, 2018–November 26th, 2018), all goats fed a corn-soybean meal basal (CON) diet with 600 g/d dry matter intake (DMI). Albendazole and Lvermectin Premix (0.1 g/kg BW) was mixed into the feed for expelling parasite on November 3th, 2018. In the adaptation stage following the preparation period, experimental goats were randomly allotted to CON diet and replacing soybean meal with 100% of corn (CRS) by body weight for 21 days (19.83 ± 0.53 kg; about 8 ± 0.3 months old; 12 replicates/group). Formal trial following adaptation, two groups were fed CON and CRS diet respectively for an additional 36 days, about 11 ± 0.2 months old at the end of the trial. All the goats are weighed at the beginning and end of the formal trial. The ingredients and nutrient composition of the two diets are shown in Table 1. The dietary neutral detergent fiber (NDF) and ash-free acid detergent fiber (ADF) were determined according to the methods of Van Soest, Robertson & Lewis (1991), while dry matter (DM), crude ash (ASH), crude protein (CP), and starch analyses were performed as outlined by the AOAC (1990). Gross energy content was measured using an adiabatic bomb calorimeter (IKA, Staufen, Germany). The dietary crude protein content of the CON group was 10.77% and the protein restricted group was 5.52%. All animals were fed with equal portion (600 g/d DMI) twice per day at 07:00 and 17:00 with a 30:70 concentrate-to-forage ratio, and had free access to water. The forage is the same batch of dry straw. Four goats were excluded from our study due to pregnancy, and eventually total twenty goats (CON = 11; CRS = 9) were anesthetized by an intravenous overdose of sodium pentobarbital and slaughtered after overnight fasting for sampling.

**Table 1:** Nutritional composition of the experimental diets.

	CON group	CRS group
TMR feed composition, %		
Ricestraw	70	70
Soybeans	15	0
Corn	8.2	23
Wheatbran	2.9	2.9
CaCO3	0.1	0.1
CaH2PO4	0.3	0.5
Fat	1	1
Nacl	0.5	0.5
Premix	2	2
TMR nutritional level		
DM (%)	96.2	95.9
ASH (%)	12.71	12.31
CP (%)	10.8	5.5
NDF (%)	49.8	50.9
ADF (%)	28.4	28.9
Starch (%)	11.5	20.7
Energy (MJ/Kg)	16.8	16.9

**Note:**

CON group means control group; CRS group means substitution of soybean meal with corn group; Premix (per kilogram) contains 6.9 g Fe, 4.4 g Cu, 1.1 g Co, 11.2 g I, 11.0 g Mn, 4.6 g Zn, 0.3 g Se, 104.2 g Mg, 10,000,000 IU vitamin A, 16,000,000 IU vitamin D, 12,000 IU vitamin E, 400 g NaHCO3, and 400.9 g carrier.

### Sample collection and index detection

Blood sampling was performed at the same time in the day 1, 4, 7, 16 and 36 of experiment period at 8:00 am in order to minimize the influence of circadian rhythms (Piccione & Caola, 2002). A portion of the samples were injected into tubes containing EDTA and were delivered to the laboratory immediately for determinations of blood cell counts using an automated cell counter (Countess II; Thermo Fisher Scientific Co., Ltd., shanghai, China): white blood cell count (WBC), neutrophil count (Neu), lymphocyte count (Lym), monocyte count (Mon), eosinophil count (Eos), basophils count (Bas). Another portion of the samples were subsequently placed at room temperature for 30 min, before the final centrifugation was operated at 1,500 × g for 10 min at 4 °C. The isolated serum samples were instantly frozen in liquid nitrogen and stored at −80 °C until analysis. Due to financial reasons, only serum samples in the day 7 and 36 were used to detect the level of anti-inflammatory cytokines (IL-4, IL-5 and IL-13) and antibodies (IgG, IgA and IgM) using commercial goat ELISA kits respectively (Jiangsu Meimian industrial Co., Ltd., Jiangsu, China). Kit numbers: MM-0099O2 (IL-4), MM-1752O2 (IL-5), MM-1753O2 (IL-13), MM-35250O2 (IgG), MM-1290O2 (IgA), MM-1289O2 (IgM).

The duodenum, jejunum, ileum and colon segments were collected and washed with ice-cold 0.9% sodium chloride solution. Then samples were cut into 0.4 × 0.4 cm sections and immediately frozen in liquid nitrogen and stored at −80 °C for subsequent RNA extraction. The primer sequences of the TLR4 pathway-related genes (TLR4, MyD88, TRAF6, IFN-β, TNF-α, IL-12B, NLRP3, IRF3, IL-1β, TBK1, NF-κB, TIRAP, IL-18, AP-1, MAPK1 and IRAK1) and hybridization positions on the sequence were summarized in Table S1 and File S1, respectively. Total RNA from the duodenum, jejunum, ileum, and colon were extracted using AG21017 (Accurate Biology, Changsha, China) following the manufacturer’s directions. RNA concentration and its purity were measured using a NanoDrop 2000 spectrophotometer (Thermo Fisher Scientific, Waltham, MA, USA). Agarose gel electrophoresis of total RNA were used to check the integrity of RNA. A total amount of 1 μg RNA per sample was used to produce cDNA by using a PrimeScript™ RT reagent kit (Accurate Biology, Changsha, China). Real-time qPCR for the expression of target genes was performed using a SYBR Premix Ex Taq II 98 (Tli RNaseH Plus) detection kit (Accurate Biotechnology, Changsha, China). Reactions were performed on a Lightcycler 480 II System (Roche, Basel, Switzerland). The thermal cycling parameters were as follows: 95 °C for 10 min for initial denaturation, and then cycled at 95 °C for 20 s and 60 °C for 1 min for 40 cycles of amplification. The relative expression of a gene was calculated relative to the mean expression of jejunum in the control group using the ∆∆Ct method (Schmittgen & Livak, 2008), with Glyceraldehyde-3-phosphate dehydrogenase (GAPDH) is a classic internal gene, which has been tested across all tissue samples. In this study, GAPDH showed a very stable state. Other researchers also used GAPDH as an internal control in goats (He et al., 2012; Sun et al., 2013; Sun et al., 2017), cattle (Ishida et al., 2013; Wang et al., 2017), yak (Pan et al., 2015; Qin et al., 2015), sheep (Sun et al., 2019; Liu et al., 2017).

### Data analysis

All samples were run in triplicate to increase the credibility of the results. The assumptions of homogeneity and normality of variances of all data were checked using the UNIVARIATE and PLOT procedures (ver. 9.4; SAS Institute Inc., Cary, NC, USA). The non-normally distributed variables were then converted through logarithmic transformation. The variables representing the immune function of goats (WBC, Neu, Lym, Mon, Eos, Bas, IgG, IgA and IgM) were subsequently analyzed using a General Linear Model of IBM SPSS Statistics Version 21. The statistical model involved diet, sampling date, and their interaction as the fixed effects, with sampling date as the repeated measurement and individual animal as the experimental unit. The model used for the analysis was as follows:

$$\rm {Y} = {\mu} + D_i + T_j + DT_{ij}+ \epsilon {ij},$$

where Y is the dependent variable, μ is the population mean for the variable, Di is diet (i = control, low-protein diet) as the fixed effect, Tj is sampling date (j = day 1, 4, 7, 16, 36) as the fixed effect, DTij is the interaction between diet and sampling date, εij is the random error associated with the observation of ij.

RT-PCR data were subsequently analyzed through a PROC MIXED model (ver. 9.4; SAS Institute Inc., Cary, NC, USA), in which the diet, intestinal region and their interaction as the fixed effects, with individual animal as the experimental unit (degrees of freedom was 57; CON, *n* = 11; CRS group, *n* = 8). Tukey test was used in the results. The model used for the analysis was as follows:

$$\rm {Y} = {\mu} + D_i + R_j + DR_{ij}+ G_{ik}+\epsilon {ijk},$$

where Y is the dependent variable, μ is the population mean for the variable, Di is diet (i = control, low-protein diet) as the fixed effect, Rj is intestinal region (j = duodenum, jejunum, ileum, colon) as the fixed effect, DRij is the interaction between diet and intestinal region, Gik is growing goat (k = 1,2,3…12) as the random effect and εijk is the random error associated with the observation of ijk.

Statistical significance was declared at *P*-value < 0.05, and *P*-value between 0.05 and 0.10 was considered trending towards significance. All the results were expressed as mean ± standard error of the mean.

## Results

### Effects of a CRS diet on the immune status of growing goats

The haematology results are shown in Table 2, the CRS group had higher Bas (*P* = 0.04) than that from CON group. However, the WBC, Neu, Lym, Mon and Eos were similar in the two group.

**Table 2:** Effect of substitution of soybean meal with corn on blood cell counts.

Item	CON group					CRS group							
	Day1	Day4	Day7	Day16	Day36	Day1	Day4	Day7	Day16	Day36	*P*_treat_	*P*_time_	*P*_treat*time_
WBC, 10^9/L	13.76 ± 0.78	13.98 ± 0.75	12.95 ± 0.72	14.31 ± 0.83	13.45 ± 0.78	17.43 ± 1.52	14.89 ± 1.02	15.02 ± 0.92	13.99 ± 0.88	14.72 ± 0.95	0.15	0.96	0.47
Neu, 10^9/L	6.87 ± 0.50	6.88 ± 0.55	5.95 ± 0.45	7.91 ± 0.80	6.99 ± 0.73	9.91 ± 1.29	7.16 ± 0.73	7.43 ± 0.86	6.66 ± 0.66	8.53 ± 0.75	0.17	0.06	0.01
Lym, 10^9/L	6.29 ± 0.44	6.35 ± 0.54	6.38 ± 0.58	5.79 ± 0.43	5.34 ± 0.50	6.72 ± 0.38	7.02 ± 0.51	6.76 ± 0.44	6.24 ± 0.45	5.14 ± 0.37	0.58	<0.01	0.37
Mon, 10^9/L	0.29 ± 0.03	0.43 ± 0.09	0.34 ± 0.03	0.30 ± 0.03	0.76 ± 0.18	0.38 ± 0.05	0.33 ± 0.05	0.38 ± 0.06	0.37 ± 0.05	0.63 ± 0.16	0.93	0.11	0.48
Eos, 10^9/L	0.17 ± 0.04	0.18 ± 0.03	0.16 ± 0.03	0.15 ± 0.02	0.22 ± 0.004	0.20 ± 0.04	0.22 ± 0.08	0.29 ± 0.12	0.54 ± 0.30	0.26 ± 0.09	0.27	0.23	0.14
Bas, 10^9/L	0.15 ± 0.02	0.14 ± 0.02	0.12 ± 0.01	0.16 ± 0.01	0.14 ± 0.01	0.22 ± 0.02	0.17 ± 0.01	0.17 ± 0.02	0.18 ± 0.02	0.16 ± 0.01	0.04	<0.01	0.08

**Note:**

CON group means control group; CRS group means substitution of soybean meal with corn group; the actual sample size was same between CON (*n* = 11) and CRS group (*n* = 9).

No significant difference in concentration of inflammatory cytokine (IL-4, IL-5 and IL-13) or immunoglobulins (IgG, IgA and IgM) was observed between two groups (Table 3).

**Table 3:** Effect of substitution of soybean meal with corn on the production of anti-inflammatory cytokines and antibodies.

Item	CON group		CRS group				
	Day7	Day36	Day7	Day36	*P*_treat_	*P*_time_	*P*_treat*ime_
IL4, pg/mL	45.48 ± 2.99	53.88 ± 2.07	48.81 ± 2.62	58.98 ± 2.19	0.081	0.004	0.752
IL5, pg/mL	530.03 ± 25.95	620.98 ± 26.97	530.60 ± 34.35	566.49 ± 31.07	0.359	0.050	0.373
IL13, pg/mL	365.41 ± 16.76	411.27 ± 21.72	373.42 ± 27.31	436.54 ± 14.01	0.392	0.023	0.700
IgG, μg/mL	320.38 ± 32.48	518.75 ± 28.57	335.83 ± 16.86	515.88 ± 24.59	0.836	<0.01	0.762
IgM, μg/mL	1517.19 ± 102.17	2031.96 ± 83.95	1625.13 ± 119.36	2089.64 ± 81.49	0.448	<0.01	0.780
IgA, μg/mL	173.54 ± 15.92	231.34 ± 13.48	201.55 ± 19.09	228.96 ± 8.16	0.412	0.009	0.308

**Note:**

CON group means control group; CRS group means substitution of soybean meal with corn group; the actual sample size was different between CON (*n* = 11) and CRS group (*n* = 9) due to hemolysis.

### Effects of a CRS diet on the expression of genes related to TLR4 pathway in the gut of growing goats

Agarose gel electrophoresis of total RNA extracted from gut samples (*n* = 8/per group) were showed in Fig. S1. The results showed that the integrity of RNA met the experimental requirements. The efficiency of primers calculated from the slope (between −3.19 and −3.45) was between 94.92% and 105.69%. The above results indicated that the amplification efficiency of primers was good. The stability of reference gene in the experiment has been tested across all tissue samples. The results showed that GAPDH was a classic internal gene (Fig. S2).

**Figure 1:**
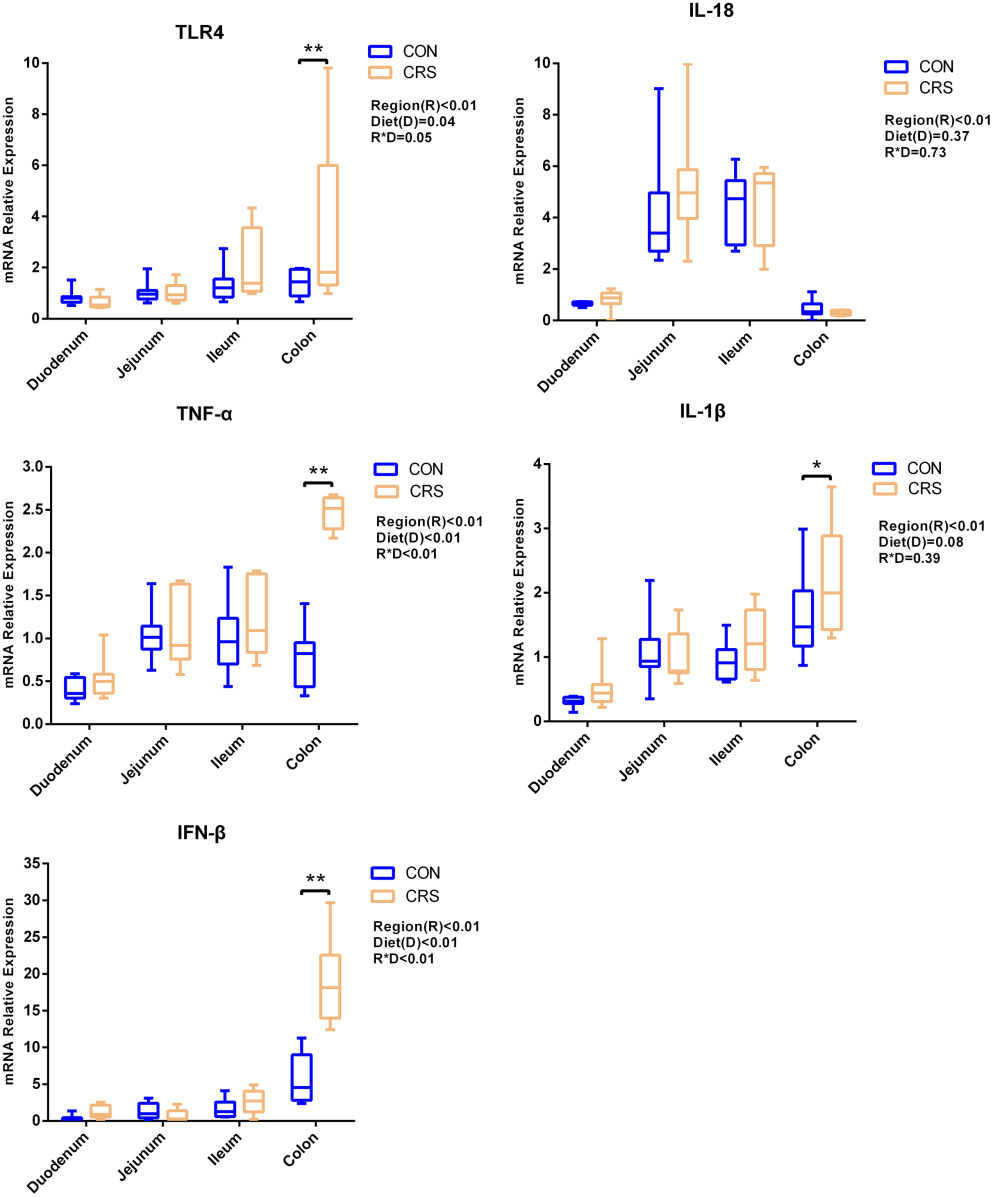
The change of relative gene expression levels of TLR4 and cytokines between CON (*n* = 11) and CRS groups (*n* = 8). *p < 0.05; **p < 0.01.

**Figure 2:**
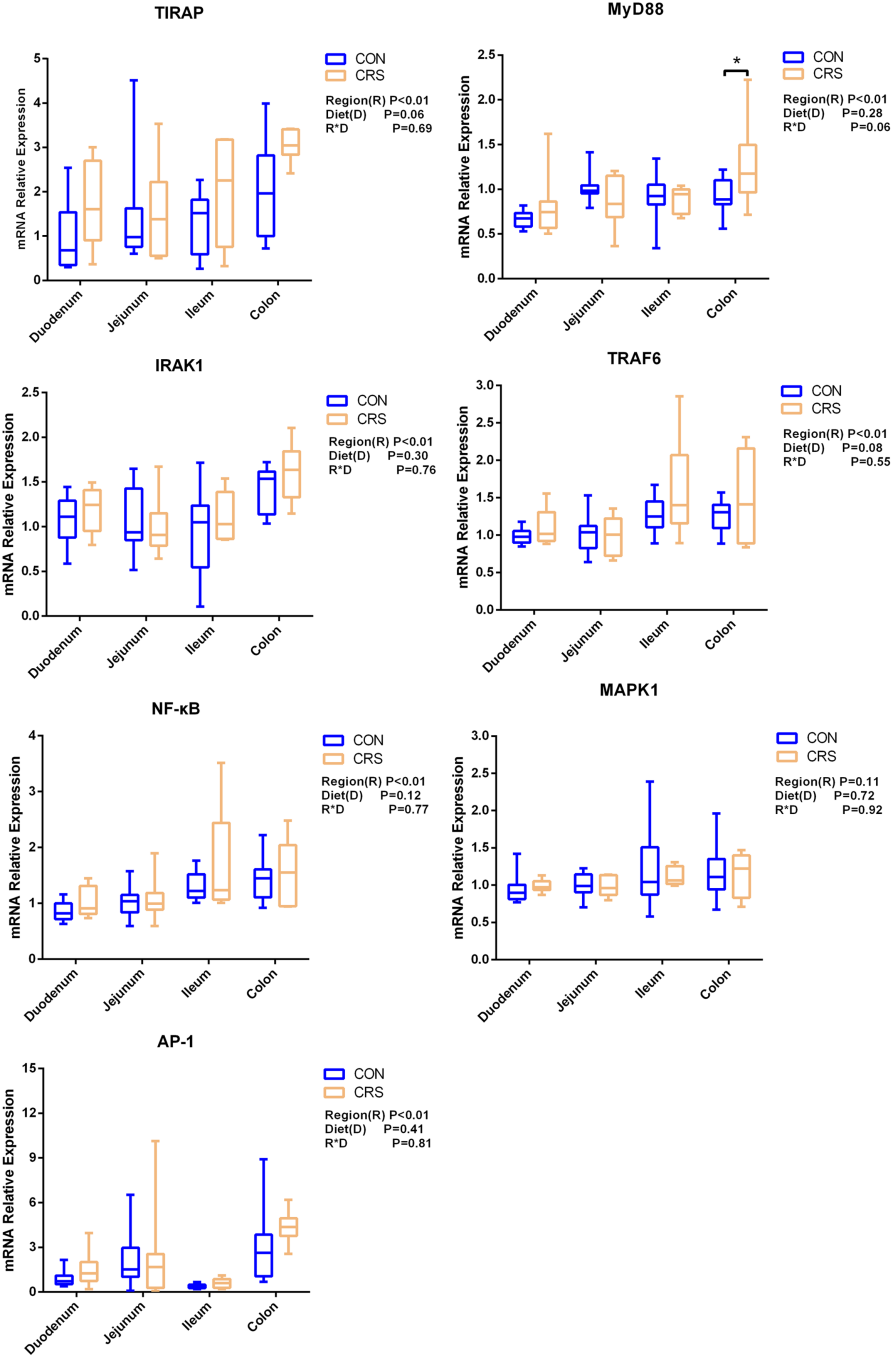
The change of relative gene expression levels of MyD88-dependent pathway between CON (*n* = 11) and CRS groups (*n* = 8). *p < 0.05.

**Figure 3:**
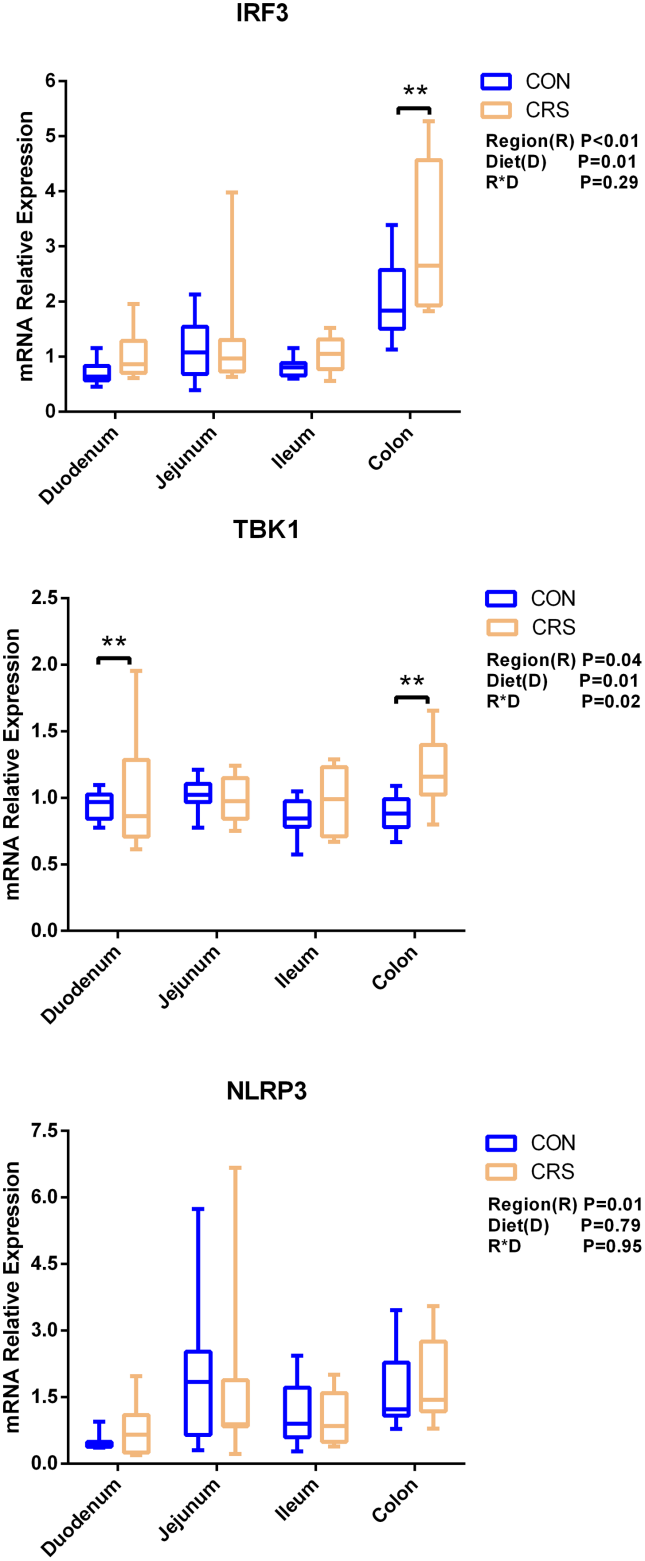
The change of relative gene expression levels of MyD88-independent pathway between CON (*n* = 11) and CRS groups (*n* = 8). **p < 0.01.

The mRNA expression of inflammatory cytokines in duodenum, jejunum, ileum and colon were shown in Fig. 1. Expression of TLR4 (*P* = 0.001), TNF-α (*P* < 0.001), IFN-β (*P* < 0.001) and IL-1β (*P* = 0.041) in colon were significantly up-regulated in growing goats fed a CRS diet. As shown in Fig. 2, the mRNA expression of the key nodes (TIRAP, IRAK1, TRAF6, NF-κB, MAPK1 and AP-1) in the TLR4/MyD88-dependent pathway were not altered by a CRS diet even though the expression of MyD88 was significantly up-regulated (*P* = 0.019) in the colon. For the TLR4/MyD88-independent pathway, the expression of TBK1 was significantly up-regulated in the duodenum (*P* = 0.001) and colon (*P* = 0.005), and the expression of IRF3 (*P* = 0.004) was significantly up-regulated only in the colon (Fig. 3).

## Discussion

In the present study, there was no difference in final body weight of goats between the CON (19.81 ± 0.78 kg) and CRS groups (19.97 ± 0.62 kg), while the crude protein intake in the CON group (59.51 ± 1.37 g/d) was significantly higher than that in the CRS group (28.95 ± 0.78 g/d) and the percentage of protein restriction is approaching to 49% (unpublished data). It has been reported that protein-malnourished mice were leukopenic, with reduced numbers of neutrophils, lymphocytes and monocytes (Mello et al., 2014). In our study, the concentration of Bas was increased in CRS, which was unexpected. Similarly, a previous study has shown that an increase in the count of circulating neutrophils was observed in cattle fed a high starch diet (Francesio et al., 2020). It is possible that the high level of dietary starch may be a factor resulting in the increased concentration of Bas. Previous studies have shown that carbohydrates could regulate the production of pro-inflammatory cytokines by inducing expression of genes of inflammatory molecules and regulating innate and adaptive immunity (Braun & Duvillard, 2004; Xu, Liu & Komai-Koma, 2004). Since bacterial endotoxins derived from gram-negative bacteria accumulate in the digestive tract especially in the hindgut of ruminants fed a high concentrate diet (Khafipour, Krause & Plaizier, 2009; Tao et al., 2014), the high starch in CRS may result in more endotoxins and therefore inducing increased Bas.

In our study, the CRS failed to reduce specific immunoglobulin (IgA, IgM and IgG) production, which was consistent with the results reported by Kefyalew et al. (2021) who elucidated that reducing dietary protein levels from 14.5% to 12.5% of DM did not affect the formation of circulating total immunoglobin concentration and specific antibodies (IgA and IgM) in growing lambs. In addition, our results showed that the protein-restricted diet had no effect on IL-4, IL-5 or IL-13 level in serum. In contrast, a study using goats as experimental animals has shown that maternal protein restriction during late gestation decreased plasma IgG and IgM concentrations of neonatal goat kids even though there were no differences in any plasma immune parameters at 6 weeks of age (He et al., 2014). Another experimental studies in both animals and humans have shown that sufficiently severe protein deficiency would interfere with antibody formation and increase the susceptibility to disease (Scrimshaw, 2007; Tang et al., 2018). The degree of severity of malnutrition might be another factor that results in the variable immunoglobulin concentrations in malnourished subjects (Gross & Newberne, 1980).

Li et al., 2014 have noticed that a TLR4 signaling pathway was required for recombinant *Brucella abortus* BCSP31-induced Th1 immune response *in vivo* and *in vitro*. Pro-inflammatory genes (TNF-α, IFN-β and IL-1β) are the downstream signals of TLR-4, and the expression of TLR4, TNF-α (Th1 cytokine) and IFN-β in the colon of CRS group were significantly up-regulated while the expression of IL-1β (Th1 cytokine) in the colon tends to be increased in the CRS group compared with the CON group. It suggests that CRS diet might activate the TLR4 pathway and promote the secretion of intestinal inflammatory cytokines. Based on the results that a low protein diet may increase the mRNA expression of Th1 cytokine and it appears that immune modulation is at a local level opposed to systemic. Interestingly, few changes in the genes related to TLR4/MyD88-dependent pathway was observed induced by the CRS diet, whereas the expression of the key nodes in the TLR4/MyD88-independent pathway, such as IRF3 and TBK1, were up-regulated. In agreement with our results, a previous study has shown that the occurrence of inflammation in sheep epithelial cells following capsular polysaccharide and *M. ovipneumoniae* treatment was linked to a MyD88-independent signaling pathway (Jiang et al., 2017). Furthermore, it has been reported that MyD88-independent signaling accounts for most of the LPS response (Vaure & Liu, 2014). In agreement with this notion, the CRS diet induced the release of a pro-inflammatory cytokine such as IL-1β, TNF-α and IFN-β mainly through a MyD88-independent pathway, and colon may be the easiest targeted section in the intestinal tract. A limitation of this study was that the higher starch content in the low protein diets which might influence the results. The intestinal immune system was sensitive to these challenges. It has to be considered that the low protein content and the high starch content might be factors, which influenced the present results. Therefore, further studies should focus on the relationships between low-protein, high-starch diet and intestinal immune mechanisms to facilitate the animal health. From a broader point of view, the findings of the present study are of importance for humans as well, especially for children. People and children in underdeveloped countries of the world might not be able to consume a sufficient amount of dietary protein whereas low-priced carbohydrates are available. As of 2019, there were 149 million stunted under-four children and 50 million emaciated children worldwide (https://www.unicef.org/reports/state-of-worlds-children-2019). According to a recent study children die of severe deficits of dietary protein and energy account for 11.4% of deaths under the age of 5 years (Black, Victora & Walker, 2013). Although the potential mechanism of CRS diet in regulating the production of proinflammatory cytokines remains largely unknown, our findings using goats as experimental animals may provide a better understanding on how malnutrition affecting the immune system for humans especially for children.

## Conclusion

In this study, the immune system in growing goats was not profoundly changed by the CRS diet. Nevertheless, the expression of pro-inflammatory cytokines TNF-α (Th1 cytokine) and IFN-β were significantly increased and IL-1β (Th1 cytokine) was tended to be increased in colon of goats fed the CRS diet. Moreover, the CRS diet upregulated the expression of TBK1 and IRF3 in the TLR4/MyD88-independent pathway in the colon. Thus, the CRS diet may induce a proinflammatory response through a TLR4-MyD88-independent pathway and colon may be the easiest targeted section in the intestinal tract. The immune modulation induced by a CRS diet was at a local level opposed to systemic.

## Supplemental Information

10.7717/peerj.12910/supp-1 Supplemental Information 1 Agarose gel electrophoresis of total RNA extracted from gut samples.

10.7717/peerj.12910/supp-2 Supplemental Information 2 Agarose gel electrophoresis of reference gene in gut samples.

10.7717/peerj.12910/supp-3 Supplemental Information 3 RT-PCR primers of genes related to TLR4 pathway.

10.7717/peerj.12910/supp-4 Supplemental Information 4 The hybridization positions of primers on the sequence.

10.7717/peerj.12910/supp-5 Supplemental Information 5 Raw data.

10.7717/peerj.12910/supp-6 Supplemental Information 6 Author Checklist.

## Abbreviations

WBC: white blood cell
Neu: neutrophil
Lym: lymphocyte
Mon: monocyte
Eos: eosinophil
Bas: basophils
IL-4: interleukin-4
IL-5: interleukin-5
IL-13: interleukin-13
IgG: immunoglobin G
IgA: immunoglobin A
IgM: immunoglobin M
GAPDH: glyceraldehyde-3-phosphate dehydrogenase
MyD88: myeloid differentiation factor 88
TLR4: toll-like receptor 4
TRAF6: TNF receptor-associated factor-6
IFN-β: interferon -β
IL-1β: interleukin-1 beta
TNF-α: tumour necrosis factor-α
IL-6: interleukin-6
NLRP3: pyrin domain-containing 3
IRF3: interferon regulatory factor 3
TBK1: TANK-binding kinase-1
NF-κB: NF kappa B
IL-18: interleukin-18
TIRAP: toll-interleukin 1 receptor (TIR) domain-containing adapter protein
IRAK1: interleukin-1 receptor activated kinase 1
MAPK1: mitogen-activated protein kinase 1
AP-1: activating protein-1.

## Additional Information and Declarations

### Data Availability

The following information was supplied regarding data availability: The raw data is available in the Supplemental File.
