# Supplementary figures and images for: Effects of substituting soybean meal with corn on immune function and gene expression of gut TLR4 pathway of growing goats
Source: PeerJ. 2022 Feb 7;10:e12910. doi: 10.7717/peerj.12910 (PMC8830315; doi:10.7717/peerj.12910)

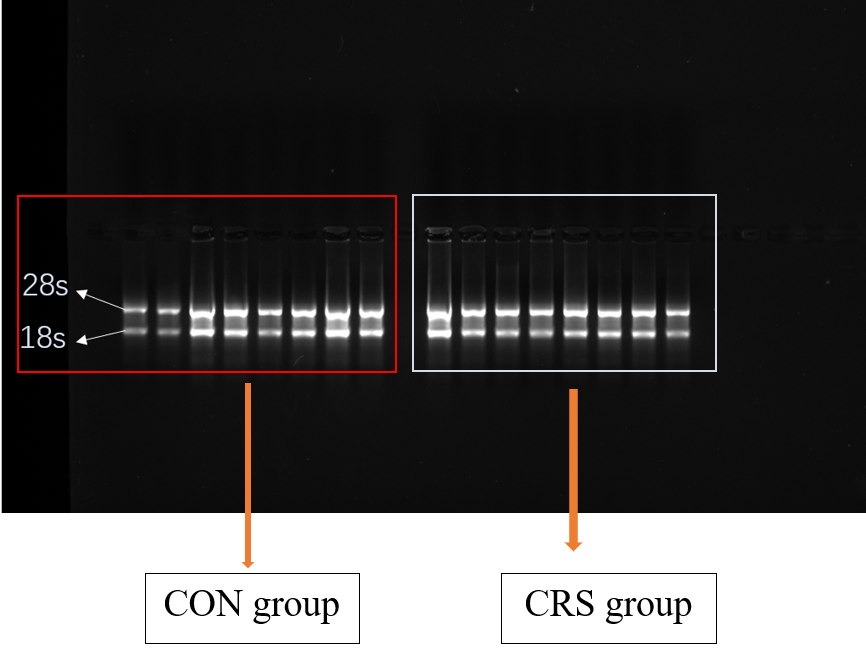

Supplement: Supplemental Information 1 [file peerj-10-12910-s001.png]

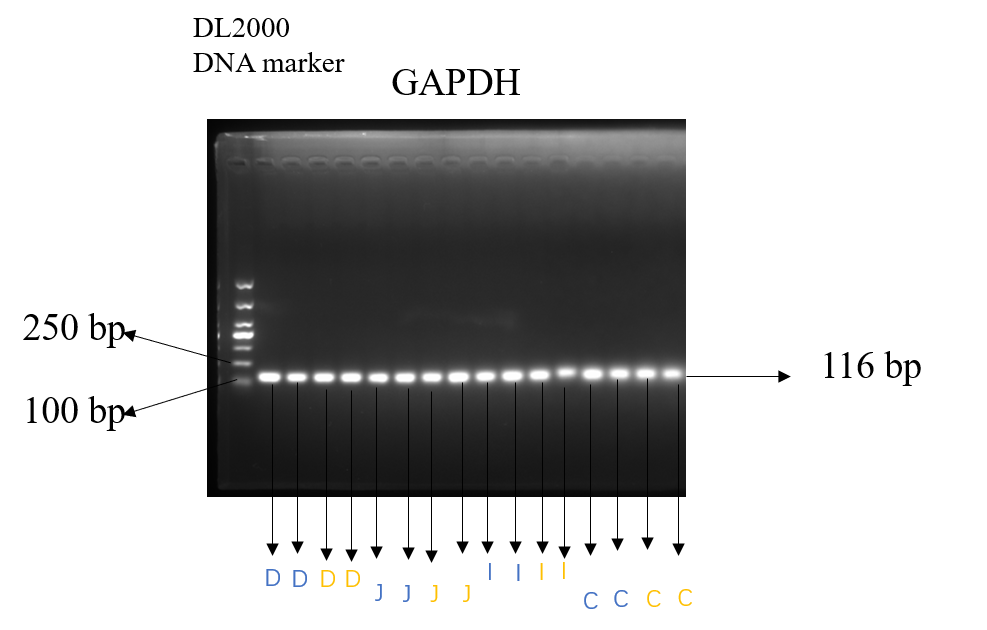

Supplement: Supplemental Information 2 [file peerj-10-12910-s002.png]
