# Supplementary material for: Effects of substituting soybean meal with corn on immune function and gene expression of gut TLR4 pathway of growing goats
Source: PeerJ. 2022 Feb 7;10:e12910. doi: 10.7717/peerj.12910 (PMC8830315; doi:10.7717/peerj.12910)
Supplement: Supplemental Information 3 [file peerj-10-12910-s003.docx]

**Supplementary information**

**Table S1** RT-PCR primers of genes related to TLR4 pathway.

| Gene Name | Sequence (5' to 3') | Product size | Accession number |
| --- | --- | --- | --- |
| TLR4 | GTCAAGGACCAGAGGCA | 114 bp | NM001285574.1 |
|  | GCTCATCTGACAAGTGGC |  |  |
| MyD88 | TGCCTTCATCTGCTACT | 186 bp | JQ308783.1 |
|  | GAGACAACCACCACCAT |  |  |
| TRAF6 | CCGTGCACATTCAGTGCTTT | 86 bp | JQ308791.1 |
|  | TGCGACTGGGTGTTCTCTTG |  |  |
| IFN-β | GGAAGATGCCGTATTGGT | 157 bp | JX458085.1 |
|  | TTCCTTCTGGATTGGCTC |  |  |
| TNF-α | CCACTGACGGGCTTTACCT | 141 bp | AY304502.1 |
|  | TGATGGCAGAGAGGATGTTG |  |  |
| IL-12B | ATTGAGGTCGTGATGGAAGC | 190 bp | NM001285700.1 |
|  | GGGAGAAGTAGGAATGTGGG |  |  |
| NLRP3 | GTCCGTTTCCTCTTTGG | 148 bp | XM005682796.3 |
|  | TGGGCTCAGTCTGTAGTGT |  |  |
| IRF3 | AAGTGTTGCGTTTAGCGG | 156 bp | KU182749.1 |
|  | GCACAATGTCTTCCTGGGT |  |  |
| IL-1β | AAGGCTCTCCACCTCCTCTC | 114 bp | DQ837160.1 |
|  | TTGTCCCTGATACCCAAGG |  |  |
| TBK1 | AGACATACGCACCAAGC | 105 bp | KU182750.1 |
|  | TCGGCAAGTAATCCACC |  |  |
| NF-κB | CAGCTCACAGATCGGGAAAAG | 115 bp | JQ342088.1 |
|  | CGGTGCTGTCTGGAAGGAA |  |  |
| TIRAP | GGCAAGATGGCTGACTGG | 165 bp | MN871405 |
|  | AGGCGACGGTGTAGGGA |  |  |
| IL-18 | AAATGGCGACCTGGAAT | 97 bp | NM001285544.1 |
|  | TCCCTGGCTAATGAAGA |  |  |
| AP-1 | CAGACGGTGCCCGAGAT | 228 bp | JQ937364.1 |
|  | CACCTGTTCCCTGAGCATA |  |  |
| MAPK1 | AGGGTTCCTGACCGAGTA | 105 bp | NM001314202.1 |
|  | CAGCCCACGGACCAAAT |  |  |
| IRAK1 | GGGCAGTGATGAGGAACA | 154 bp | MN871406 |
|  | GCACAGTAGCCAGCAAAGT |  |  |
| GAPDH | TTCCACGGCACAGTCAAG | 116 bp | AJ431207.1 |
|  | TACTCAGCACCAGCATCACC |  |  |
