# Supplementary material for: Effects of substituting soybean meal with corn on immune function and gene expression of gut TLR4 pathway of growing goats
Source: PeerJ. 2022 Feb 7;10:e12910. doi: 10.7717/peerj.12910 (PMC8830315; doi:10.7717/peerj.12910)
Supplement: Supplemental Information 4 [file peerj-10-12910-s004.docx]

Green represents the Forward primer while yellow represents the Reverse primer

TLR4

ATGATGGCTCGTGCCCGCCGGGCTGCGGCTCTGATCCCGGCCATGGCCATCCTCTCCTGCCTGAGAACCGAGAGCTGGGACCCTTGCGTCCAGGTTGTTCCTAACATCAGTTACCAATGCATGGAGCTGAATCTCTACAAAATCCCCGACAACATCCCCGTATCAACCGAGATGCTGGACCTGAGCTTTAACTACCTGAGACATTTAGGCAGCCATAACTTCTCCAGGTTCCCAGAACTGCAAGTGCTGGATTTATCCAGATGCGAAATTAAGATTATTGAAGACAACACATTTCAAGGCCTAAACCACCTCTCCACCTTGATACTGACGGGAAACCCTATCCAGAGTTTAGCCTGGGGAGCCTTTTCTGGGCTATCAAGTTTACAGAAGCTGGTGGCCGTGGAGACAAACCTAGTATCTCTAGATGACTTCCCCATTGGACATCTCAAAACCTTGAAAGAGCTTAATGTGGCTCACAATTTTATCCATTCCTTCAAGTTACCTGAATATTTTTCTAACCTTCCCAACCTGGAGCACTTGGATCTTTCTAACAACAAAATCCAAAATATTTATTATGAAGATGTGAAAGTTCTACATCAAATGCCCCTACTCAACCTCTCTTTAGATTTGTCCCTGAACCCTTTAGAATTTATTGAACCAGGTACCTTTAAAGAAATTAAGCTCAATGGATTGACTCTGCGAAGTAATTTTAACAGTTCATATGTCATGAAAACTTGTATTCAAGGTCTGGCTGGTTTAAAAATCAACCGGCTGGTTTTGGGAGAATTTAAAAATGAAAGGAAGTTGCAAAAATTTGACAGATCTTGCCTGGAGGGACTGTGCAACCTGACCATTGAGCAATTCCGGATAGCGTACTTGAACAAATTCTCACGGAACGATACAGACTTATTTAATTGTTTGGCAAATGTTTCTATGATTTCTCTGTTGAGTATACCTTTAGGAAGTCTACAAGCCCTTCTTAAAGATTTTAGATGGCAACACTTAGAAATGATTAACTGTGACTTTGATAAGTTTCCTGCACTGGAGCTCCGTTCTCTCAAAAAGTTTGTTTTCACAGACAACAAAGATGTAAGCAGTTTTACTAAGACTGAGCTACCAAGCCTTCAGTATCTAGATCTCAAAAGAAATCACTTGAGTTTCAAGACCTGCTGTTCTCACACTGATTTTGGGACAACCAACCTGAAGCATTTAGATCTGAGCTTCAATGACGTCATTACCTTAAGTTCAAACTTCATGGGCTTAGAGCAGCTAGAACACCTGGATTTTCAGCATTCCACTCTGAAACAGATCAATGCTTTTTCAACGTTCCTATCACTCAGAAACCTCCGCTACCTTGACATCTCTTACACCAACATCCGGATTGTCTTCCACGGCATCTTTACTGGCTTAGTCAGTCTGCAGACCTTGAAAATGGCAGGCAACTCTTTTCAGAACAACTTGCTCCCTGACATCTTCACAGAGCTGACTAACTTAACCATCTTGGACCTCTCTAAGTGTCAACTGGAACAGGTGTCCTGGACGGCATTTCACTCCCTCCCTAGCCTTCAGGTGCTGAATATGAGTCACAACAAACTCTTGTCATTGGATACATTTCTTTATGAACCACTCCACTCGCTCCGGATCCTAGACTGCAGTTTCAACCGTATCACGGCCTCTAAGGAGCAAGAACTACGGAGTTTGCCAAGGAACCTCACTTGGCTAAATCTTACTCAGAATGAATTTGCTTGTGTTTGTGAACATCAGAGTTTCCTGCAGTGGGTCAAGGACCAGAGGCAGCTCTTGGTGGGAGCTGAGCAAATGATGTGTGCAGAGCCTTTAGATGTGAAGGACATGCCAGTGCTTGGTTTCAGGAATGCCACTTGTCAGATGAGCAAGACGATCATCAGCGTGTCGGTTGTCACTGTACTCCTGGTATCTGTGGTAGGAGTCCTAGTCTATAAGTTCTATTTCCACCTGATGCTTCTTGCTGGCTGCAAAAAGTATGGCAGAGGTGAAAGCACCTATGATGCCTTTGTAATCTACTCGAGCCAGGATGAAGCCTGGGTGCGGAATGAACTGGTAAAGAACTTGGAGGAGGGCGTGCCCCCCTTTCAGCTCTGCCTTCACTACAGGGACTTTATTCCTGGGGTGGCCATCGCCGCCAACATCATCCAGGAAGGTTTCCACAAGAGCCGTAAGGTGATTGTCGTGGTGTCCCAGCACTTCATCCAGAGCCGATGGTGTATCTTCGAGTATGAGATTGCCCAGACCTGGCAGTTTCTGAGCAGCCGTGCTGGCATCATCTTCATCGTCCTGCAGAAGCTGGAGAAGTCTCTCCTGCGGCAGCAGGTGGAACTCTATCGCCTTCTGAACAGGAACACCTACCTGGAGTGGGAGGACAGTGTCCTGGGGCGGCATGTCTTCTGGAGAAGACTCAGAAAAGCCTTGCTGGCTGGTAAACCCCGGAGTCCAGAAGGAACAGCAGATGCAGAGACCAACCCGCAGGAAGCGACCACCTCCACCTGA

MyD88

AGCAGCATAACTCGGATAAATGACATGGCAGGCATCACTATTCGCGACGACCCCCTAGGGCAAAAGCCTGAGTATTTTGATGCCTTCATCTGCTACTGCCCCAGCGATATTGAGTTTGTCCACGAGATGATCCGGCAGCTGGAACAGACAAACTATCGGCTGAAGTTGTGTGTGTCTGACCGTGACGTCCTGCCTGGCACCTGTGTCTGGTCCATCGCCAGTGAACTCATTGAGAAGAGGTGCCGTCGGATGGTGGTGGTTGTCTCTGACGAATACCTGCAAAGCAAGGAATGTGACTTCCAGACTAAGTTTGCACTCAGCCTCTCTCCAGGTGCCCATCAGAAGCGACTGATCCCCATCAAGTACAAGCCAATGAAGAAAGAGTTCCCCAGCATCCTGCGCTTCATCACTGTCTGTGACTACACCAACCCCTGCACCCAGAACTGGTTCTGGACTCGCCTCGCCAAGGCCCTGTCCATGCCCTGA

TRAF6

GACGTGGAATTTGACCCACCCCTGGAAAGCAAGTACGAGTGCCCCATTTGCTTGATGGCTTTGCGGGAAGCAGTGCAGACACCTTGCGGCCACAGGTTCTGCAAAGCCTGCATCATCAAATCAATAAGGGATGCAGGTCACAAATGTCCAGTTGACAATGAAATACTTCTGGAAAATCAACTATTTCCTGACAATTTTGCAAAACGAGAGATTCTTTCTCTGATGGTGAAATGTCCAAATGAAGGTTGTTTGCACAAGATGGAACTGAGGCATCTTGAGGAGCATCAAGCACACTGTGAGTTTGCTCTTATGAGTTGTCCACAATGCCAGCGTCCCTTCCAGAAATGCCACCTTAATTTTCATATTCTCAAGGAGTGTCCAAGGAGACAGGTTCCTTGTGAAAACTGTGCTGTATTAATGGCATTTGAAGATAAAGAGATTCATGAGCAGAACTGTCCTTTGGCAAATGTCATCTGTGAATACTGCAATACCATGCTCATCAGAGAACAGATGCCCAATCACTATGATCTAGACTGTCCTACAGCCCCAGTTCCGTGCACATTCAGTGCTTTTGGTTGCCATGAAAAGATGCAGAGGAATCACTTGGCACGCCACCTGCAAGAGAACACCCAGTCGCATATGAGAATGATGGCCCAGGCTGTTCAGACTTTAAGCCTCGCAGTGGCTCCTGCACCTCAGTGCACCATGCCTCCGTATGACTCTGTCCCTCCCGCTCGGCCTTCCTCTGGGCGTCACTCAGAAGTCCACAATTTCCAAGAAACCATTCAACAGTTAGAGGGTCGCCTTGTA

IFN-β

ATGACCTACCGGTGCCTCCTCCAGATGGTTCTCCTGCTGTGTCTCTCCACCACAGCTCTTTGCAGGAGCTACAGCTTGCTTCGATTCCAACAAAGGCGGAGCACTGCGGTGTGTCAGAAACTTCTGCAGCAGTTACCTGCAACTCCTCAACATTGCCTGGAGGCCAGGATGGACTTCCAGGTCCCCGAGGAGATGAAGCAAGCACAGCAGTTCCGGAAGGAAGATGCCGTATTGGTCATGTATGAGATGCTCCAGCAGATCTTCAATATTCTCACCAGAGACTTCTCCAGCACTGGCTGGTCTGACACCATCATTGAGCACCTCCTTGAGGAACTCTATGGGCAGATGAATCGTCTGGAGCCAATCCAGAAGGAAATAATGCAGAAGCAAAACTCCACTATGGGAGACACGACCGATCTTCACCTGCAGAAATACTACTTCAACCTCGGGCAGTACCTCAAGTCCAAGGAGCACAACAGGTGTGCCTGGGCAGTCGTGCAAATGCAATTGCTCAGGATCTTTTCTTTCCTGAAGAGCCTAACAGGTTACCTCCGAAACTGA

TNF-α

ATGAGCACCAAAAGCATGATCCGGGATGTGGAGCTGGCAGAGGAGGTGCTCTCCAAGAAAGCAGGGGGCCCCCAGGGCTCCAGAAGTTGCTGGTGCCTCAGCCTCTTCTCCTTCCTCCTGGTTGCAGGAGCCACCACGCTCTTCTGCCTGCTGCACTTCGGGGTAATCGGCCCCCAGAGGGAAGAGCAGTCCCCAGCTGGCCCCTCCTTCAACAGGCCTCTGGTTCAGACACTCAGGTCATCTTCTCAAGCCTCAAGTAACAAGCCGGTAGCCCACGTTGTAGCCAACATCAGCGCTCCGGGGCAGCTCCGGTGGGGGGACTCGTATGCCAATGCCCTCAAGGCCAACGGCGTGGAGCTGAAAGACAACCAGCTGGTGGTGCCCACTGACGGGCTTTACCTCATCTACTCGCAGGTCCTCTTCAGGGGCCACGGCTGCCCTTCCACCCCCTTGTTCCTCACCCACACCATCAGCCGCATTGCAGTCTCCTACCAGACCAAGGTCAACATCCTCTCTGCCATCAAGAGCCCTTGCCACAGGGAGACCCCAGAGGGGGCTGAGGCCAAGCCCTGGTACGAACCCATCTACCAGGGAGGGGTCTTCCAGCTGGAGAAGGGAGATCGCCTCAGTGCTGAGATCAACCTGCCGGAATACCTGGACTATGCTGAGTCTGGGCAGGTCTACTTTGGGATCATCGCCCTGTGA

IL-12B

ATGCACCCTCAGCAGTTGGTCGTTTCCTGGTTTTCCCTGGTTTTGCTGGCATCTCCCATCGTGGCCATATGGGAACTGGAGAAAAATGTTTATGTTGTAGAATTGGATTGGTATCCTAATGCTCCTGGAGAAACAGTGGTCCTCACGTGTGACACTCCTGAAGAAGACGGCATCACCTGGACCTCAGACCAGAGCAGTGAGGTCCTGGGCTCTGGCAAAACCTTGACCATTCAAGTCAAAGAGTTTGGAGATGCTGGGCAGTACACCTGTCACAAAGGAGGCGAGGTTCTGAGTCGTTCACTCCTCCTGCTTCACAAAAAGGAAGATGGAATTTGGTCCACTGATATTTTAAAGGATCAGAAAGAACCCAAAGCTAAGAGTTTTTTAAAATGTGAGGCAAAGGATTATTCTGGACACTTCACCTGCTCGTGGCTGACAGCAATCAGTACTAATCTGAAATTCAGTGTCAAAAGCAGCAGAGGCTCCTCTGACCCCCGAGGGGTGACGTGCGGAGCAGCGTCACTCTCAGCAGAGAAGGTCAGCATGGACCACAGGGAGTATAACAAGTACACAGTGGAGTGTCAGGAGGGCAGTGCCTGCCCGGCCGCCGAGGAGAGCCTGCCCATTGAGGTCGTGATGGAAGCTGTGCACAAGCTCAAGTATGAAAACTACACCAGCAGCTTCTTCATCAGGGACATCATCAAACCAGACCCACCCAAGAACCTGCAACTGAGACCACTAAAGAATTCTCGGCAGGTGGAGGTCAGCTGGGAGTACCCTGACACGTGGAGCACCCCACATTCCTACTTCTCCCTGACGTTTTGTGTTCAGGTCCAGGGAAAGAACAAGAGAGAAAAGAAACTCTTCACGGACCAAACCTCAGCCAAAGTCACATGCCACAAGGATGCCAACATCCGTGTGCAAGCCCGGGACCGCTACTACAGCTCATTCTGGAGTGAATGGGCATCTGTGTCCTGCAGTTAG

NLRP3

ATGATCATGGCAAGTGTCCGCTGCAAGCTGGCTCGTTACCTGGAGGACCTGGAAGACATAGACTTTAAGAAATTCAAGATGCATTTAGAAGACTATCCCAGTCAAAAGGGCTGCACCTCAATTCCTCGGGGTCAGACAGAAAAAGCAGATCATGTGGATATAGCCACCCTGATGATTGATTTTAACGGGGAAGAGAAGGCATGGGCCATGGCCAAGTGGATTTTTGCTGCAATCAACAGGAGAGACCTTTATGAGAAAGCTAAGAGGGATGAGCCAGAATGGGAGAATACAAATGTTTCTGTGCTAAGTCAGGAAGAAAGCCTTGAAGAAGAATGGATGGGTTTACTGGGATACCTTTCCAGAATCTCTATTTGTAGAAAAAAAAAAGATTACTGTAAGAAGTACAGAAAATATGTGAGAAGCAAATTCCAGTGTATTGAAGACAGGAATGCCCGTCTGGGTGAGAGCGTGAACCTCAACAAACGCTTCACCAGGCTGCGTCTCATCAAGGAACACAGGAGCCAACAGGAGAGGGAGCATGAGCTCCTGGCCATTGGTAGGACTTGGGCCAAGATACAGGATAGCCCCGTGAGTTCCGTGAACTTGGAATTGCTGTTTGATCCTGAGGATCAGCACTCTGAGCCTGTGCACACAGTGGTGTTCCAGGGAGCAGCGGGCATTGGGAAAACAATACTGGCCAGGAAGATCATGTTGGACTGGGCCTCAGAGAAACTTTACCAGGATAGATTTGACTATTTGTTTTACATTCACTGCCGGGAGGTGAGCCTTGGGACTCAGAGGAGCCTGGGGGACCTGATCGCCAGCTGCTGCCCTGGCCCAAACCCACCCATAGGCAAGATTGTAAGCAAGTCTTCCAGGATCCTCTTCCTCATGGACGGCTTTGACGAGCTGCAAGGTGCCTTTGATGAGCACACAGAAGCACTCTGCACAAACTGGCAGAAGGTGGAGCGGGGAGACATTCTCCTGAGCAGCCTCATCAGAAAAAGACTGCTTCCTGAGGCCTCCCTCCTCATCACCACAAGACCTGTGGCCCTGGAGAAACTTCAGCACTTGCTGGGCCAGGCTCGTCATGTGGAGATCCTGGGTTTCTCAGAGGCCAGGAGGAAGGAATATTTCTTAAAGTATTTCTCAGATGAGCAGCAAGCAAGGGAAGCCTTCAGGCTGATTCAGGAGAATGAGATTCTCTTCACCATGTGCTTTATTCCTCTGGTTTGCTGGATTGTGTGCACTGGGCTGAAACAGCAGATGGATAGTGGCAAGAGTCTTGCTCGGACATCCAAGACCACCACTGCAGTGTATATCTTCTTCCTCTCCAGTTTGTTGCAATCTCAGGGAGGGAGCCAGGAGAACCACAACTCTGCTACCCTCTGGGGTCTCTGTTCATTGGCTGCAGATGGAATCTGGAACCAAAAAATCCTATTTGAGGAGTGCGATCTCAGAAATCATGGCCTGCAGAAGGCAGATGTGTCTGCTTTCTTGAGAATGAACCTGTTCCAAAAGGAAGTGGACTGCGAGAAATTCTACAGCTTCATCCACATGACTTTCCAGGAGTTCTTTGCTGCCATGTACTACCTGCTAGAAGAAGACAATCATGGGGAGATGAGGAACATGCCTCAGGCTTGTTCAAAACTTCCCAACCGAGATGTGAAGGTCCTTCTCGAAAACTATGGCAAATTCGAAAAGGGATATCTGATTTTTGTTGTCCGTTTCCTCTTTGGCCTTATAAACCAGGAGAGGACCTCCTACTTAGAGAAAAAACTGAGTTGTAAGATCTCTCAGAAAATCAGGCTAGAGCTGCTGAAATGGATTGAAGCAAAAGCCAAGGCCAAGACACTACAGACTGAGCCCAGCCAGCTGGAATTGTTCTATTGTTTGTATGAGATGCAGGAGGAGGACTTTGTGCAAAGGGCGATGGGCCATTTCCCCAAAATTGAGATCAAGCTGTCCACTAGAATGGACCATGTGGTTTCTTCTTTTTGTATTGAGAACTGTCGCCATGTGGAATCCCTTTCCCTGAGGTTGCTCCATAACTCACCCAAGGAGGAAGAAGAAGAGGAGGAAGTTAGACACTCTCATATGGACCATTCTGTTCTCTCTGATTCTGAGGTTGCATATTCTCAGGGATTGGTGAACTATTTGACCTCCAGCATTTGCAGGGGAATCTTCTCAGTCCTGAGCAATAACTGGAATCTCACTGAATTGAACCTCAGTGGCAATACCCTGGGAGACCCAGGCATGAAGGTGTTATGCGAAACACTCCAACAACCTGGCTGTAACATTCGTAGACTGTGGTTGGGACAGTGCTGCCTTTCCCATCAGTGCTGCTTCAGCATCTCCTCAGTCCTGAGCAGCAACCAGAAGCTGGTGGAACTGGACCTGAGCCACAACGCCCTGGGAGACTTCGGGATCAGACTCCTGTGTGTGGGACTGAGGCATCTGTTCTGCAATCTGAAGAAGCTCTGGTTGGTCAGTTGCTGTCTCACATCGGCAAGCTGTGAGGACCTTGCATCCGTCCTAAGCACCAACCATTCCCTGACCAGACTCTACCTGGGGGAAAATGCTCTGGGAGACTCAGGAGTTGGGATTTTGTGTGAAAAAGTAAAGAATCCACACTGTAATTTGCAGAAACTGGGGTTGGTGAATTCTGGCCTTACGTCAGGTTGTTGTCCAGCCCTATCCTCAGTGCTCAGTACAAATCAGAATCTCACCCACCTTTACCTACAGGGCAATGCTCTTGGAGACATGGGGGTCAAGCTACTCTGTGAGGGACTCTTGCACCACAACTGCAAGCTTCAAGTGTTGGAGTTAGACAACTGCAGCCTCACATCACACTGCTGCTGGGATCTTTCCACACTTCTAACTTCTAACCAGAGCCTGCGAAAGCTGAGCCTAGGCAACAACGACCTGGGTGATCTGGGGGTCATGCTTCTCTGTGAAGTGCTCAAACAGCAGGGCTGCCTCCTGAAAAGCCTGAGGTTGTGTGAAATGTATTTCAACTATGATACAAAACGTGCCCTAGAAACACTTCAAGAAGAAAAGCCTGAATTAACCATTGTCTTTGAGCCTTCTCGGTAG

IRF3

ATGGGAACCCAAAAGCCTCGGATACTGCCCTGGCTGATATCTCAGCTGGACCGAGGGGAGTTGGAGGGCGTGGCCTGGCTGGGCGAGAGCCGCACGCGTTTCCGCATCCCTTGGAAGCACGGCTTGCGGCAGGACGCCCAGCAGGAGGATTTCGGCATCTTCCAGGCCTGGGCTGAAGCCAGTGGTGCCTATACTCCTGGGAAGGATAAGCCCGACCTGCCCACATGGAAGAGGAATTTCCGGTCTGCCCTGAACCGGAAGGAAGTGTTGCGTTTAGCGGAGGACCACAGCAAGGACTCCCAAGACCCGCACAAGATCTATGAGGTTGTGAACTCAGGGGTCAGGGACATCCCTGAGACAGATACCGCTCAAGACAATGGCAGACACAGTACCTCTGATACCCAGGAAGACATTGTGCAGAAGTTACTGAGTGACATGGACTTGAGCCCAGAAGGAGGGCCCTCCAATCTGACTATGACCTCTGAGAACCCCCCTCAGCTCTTACTGAGCCCCGAATCAGACATCCCTGCTCTTTGCCCAAACTCGGGACTCTCTGAAAACCCCCTGAAGCAGCTGTTGGCGAACGAGGAAGATTGGGAGTTCGAGGTGACTGCCTTCTACCGGGGCTGTCAAGTCTTCCAGCAGACTGTCTTCTGCCCTGGGGGCCTGCGGCTGGTGGGATCAGAAGCAGGGGACAGGATGCTGCCCGGGCAGCCAATACGACTGCCGGACCCCGCAGCGTCCCTAGCAGACAAGAGCGTGGCAGACTACGTGCAGTGTGTGCTGAGCTGCCTGGGCGGGGGGCTGGCCCTGTGGCGGGCCGGGCAGTGGCTCTGCGCCCAGAGGCTGGGGCACTGCCACGTGTACTGGGCCATAGGCGAGGAACTCCTCCCCAGCTGTGGCCACAAGCCTGACGGCGAGGTCCCGAAGGACAGGGAAGGAGGTGTGTTCAACCTGGGGCCCTTCATAACAGATCTGATCAACTTCACTGAAGGAAGCAGACGTTCACCACTCTATACCCTCTGGTTCTGTGTGGGGCAGTCACGGCCCCAGGACCAGCCATGGATCAAGAGGCTTGTGATGGTCAAGGTTGTCCCCATGTGCCTCAGGGTTCTTGTAAACATAGCGCGGCAAGGGGGTGCCTCCTCCCTGGAGAACACTGTCGACCTGCACATTTCCAACAGCCAACCCCTCTCTCTCACCTCAGACCAGTACATGGCCTATCTCCAGGACCTGGCCGAGGACATGGATTTCTAG

IL-1β

ATGGCAACCGTACCTGAACCCATTAATGAAGTGATGGCTTACTACAGTGATGAGAATGAGCTGTTATTTGAGGCTGATGGCCCCAAACAGATGAAGAGCTGCACCCAACACCTGGACCTCGGCTCCATGGGAGATGGAAACATCCAGCTGCAGATTTCTCACCAGCTCTACAACAAAAGCTTCAGGCAGGTGGTGTCGGTCATCGTGGCCATGGAGAAGCTGAGGAGCCGTGCCTACGAACATGTCTTCCGTGATGATGACCTGAGGAGCATCCTTTCATTCATCTTCGAAGAAGAGCCTGTCATCTTCGAAACATCCTCCGATGAGCTTCTGTGTGACGCAGCCGTGCAGTCAGTAAAATGCAAACTCCAGGACAGAGAGCAAAAATCCCTGGTGCTGGATAGCCCATGTGTGCTGAAGGCTCTCCACCTCCTCTCACAGGAAATGAGCCGAGAAGTGGTGTTCTGCATGAGCTTCGTACAAGGAGAGGAAAGAGACAACAAGATTCCTGTGGCCTTGGGTATCAGGGACAAGAATCTATACCTGTCTTGTGTGAAAAAAGGTGATACGCCGACACTGCAGCTGGAGGAAGTAGACCCCAAAGTCTACCCCAAGAGGAATATGGAAAAGCGATTCGTCTTCTACAAGACAGAAATCAAGAACACAGTTGAATTTGAGTCTGTCCTGTACCCTAACTGGTACATCAGCACTTCTCAAATCGAAGAAAAGCCCGTCTTCCTGGGACATTTTAGAGGTGGCCAGGATATAACTGACTTCAGAATGGAAACCCTCTCTCCCTAA

TBK1

ATGCAGAGCACTTCTAATCATCTGTGGCTTTTATCTGATGTTTTAGGCCAAGGAGCTACTGCAAATGTCTTTCGTGGAAGACATAAGAAAACTGGTGACTTATTTGCTATCAAAGTATTTAATAACATAAGCTTCCTTCGTCCAGTGGATGTTCAAATGAGAGAATTTGAAGTGTTAAAAAAACTGAATCACAAAAACATTGTCAAATTATTTGCTATTGAAGAAGAGACAACAACAAGACATAAAGTACTTATTATGGAATTTTGCCCATGTGGGAGTTTATACACTGTTTTAGAAGAACCATCTAATGCCTATGGACTACCAGAGTCTGAGTTTCTAATTGTTTTGCGAGATGTGGTGGGTGGAATGAATCATCTCCGAGAGAATGGCATAGTTCACCGTGATATCAAGCCAGGAAATATCATGCGTGTGATAGGGGAAGACGGACAGTCTGTGTACAAACTCACAGATTTTGGTGCAGCTAGAGAATTAGAAGATGATGAGCAGTTTGTTTCTCTGTATGGCACAGAAGAATATTTGCATCCTGATATGTATGAGAGAGCGGTGCTAAGAAAAGATCATCAGAAGAAATACGGAGCAACTGTGGATCTTTGGAGCATTGGGGTAACATTTTACCATGCAGCTACTGGATCACTGCCATTTAGACCATTTGAAGGGCCACGTAGGAATAAGGAAGTGATGTATAAAATAATTACTGGAAAGCCTTCTGGTGCAATATCTGGAGTACAGAAAGCAGAAAATGGACCAATTGACTGGAGTGGAGACATGCCTGTTTCTTGTAGTCTTTCTCGGGGTCTTCAAGTTCTGCTTACTCCTGTTCTTGCAAACATCCTTGAGGCAGATCAGGAAAAGTGTTGGGGTTTTGACCAGTTTTTTGCGGAAACTAGTGATATACTTCACCGGATGATAGTTCATGTTTTTTCACTACAACAAATGACAGCTCATAAAATTTATATTCATGGCTATAATACTGCTACTGTATTTCATGAACTGGTATATAAACAAACCAAAATTATTTCTTCAAATCAAGAGCTTGTATATGAAGGACGACGTTTAGTTCTAGAACCTGGAAGACTGGCACAGCATTTCCCTAAAACTTCTGAGGAAAACCCTATCTTCGTAGTAAGCCGGGAACCTCTGAGTACCATAGGATTGATATATGAAAAAATTTCCCTCCCTAAAGTACATCCACGTTATGATTTAGATGGGGATGCCAGCATGGCTAAGGCAATGACAGGAGTTGTGTGTTGCGCCTGTAGAATTGCCAGTACCTTGCTGCTTTATCAGGAATTAATGCGGAAGGGGATACGATGGTTGATAGAATTAGTTAAAGATGACTATAATGAAACTGTTCACAAAAAGACAGAAGTTGTGATCACGTTGGATTTCTGCATCAGAAACATTGAAAAAACTATGAAAGTATATGAAAAGTTGATGAAGATCAACATAGAAGCAGCAGAGTTAGGTGAAATTTCAGACATACGCACCAAGCTGTTGAGACTTTCCAGTTCTCAGGGAACAATAGAAACCAGTCTCCAGGATATTGAAAGCAAATTGTCTCCAGGTGGATTACTTGCCGATACATGGACCCATCAAGAAGGCACTCATCCCAAAGATAGACATGTAGAAAAGCTACAAGTCCTGTTAAATTGCATCACAGAGATTTACTATCAGTTCAAAAAAGACAAAGCAGAACGTAGACTAGCTTATAATGAAGAACAAATCCACAAATTTGATAAGCAAAAACTGTATTATCATGCAACAAAAGCTATGACCCACTTTACGGATGAATGTGTTAAAAAGTATGAGGCTTTCTTGGATAAGTCAGAAGAATGGATGAGAAAGATGCTTCACCTTAGGAAACAGTTATTATCCCTAACTAATCAGTGTTTTGATATTGAAGAAGAAGTATCGAAGTATCAAGACTATACTAATGAGTTACAAGAAACTTTGCCACAGAAAATGTTCGTAGCCTCCAGTGGAGTCAAACATACCATGACCCCAATTTATCCATGTTCTAACACATTAGTAGAAATGACTCTTGGTATGAAGAAATTAAAGGAAGAAATGGAAGGAGTAGTTAAAGAACTTGCTGAAAATAATCATATTTTAGAAAGGTTTGGCTCATTAACCATGGATGGCGGCCTCCGCAATGTCGACTGTCTTTAG

NF-κB

GTGGGAAAACACTGTGAGGATGGCGTCTGCACTGTGACAGCTGGGCCCAAGGACATGGTGGTCGGCTTTGCAAACCTGGGTATACTTCATGTGACAAAGAAAAAAGTATTTGAAACACTGGAAGCACGAATGACAGACGCCTGTGTAAGGGGCTATAATCCCGGGCTTTTGGTGCATCCTGATCTTGCCTATTTGCAGGCAGAAGGTGGAGGAGACCGGCAGCTCACAGATCGGGAAAAGGAAATCATCCGCCAGGCAGCTCTTCAGCAGACAAAGGAGATGGACCTCAGCGTGGTACGGCTCATGTTTACAGCTTTCCTTCCAGACAGCACCGGCAGCTTCACGAGGCGTCTGGAACCCGTGGTGTCGGACGCCATCTATGACAGCAAAGCCCCCAATGCATCTAACTTGAAAATAGTAAGAATGGACAGGACAGCTGGATGTGTAACTGGAGGGGAAGAGATTTATCTTCTCTGTGACAAGGTTCAGAAAGATGACATCCAGATTCGATTTTATGAAGAGGAGGAAAATGGTGGAATTTGGGAAGGATTTGGAGATTTTTCCCCCACAGATGTTCATAGACAATTTGCCATTGTCTTCAAAACTCCAAAGTATAAAGATGTCAACATTACAAAACCAGCC

TIRAP

ATGGCATCATCAACCTCCTCCCCGGCTCCTGGCTCCAGGTCCAAGAAGCCTCTGGGCAAGATGGCTGACTGGTTCAGGCAGGCCCTGTCGAGGAAGCCCACGAAGATGCCCGCCTCCCCAGAAAGCGCCCTCAGTGACGTTTCACAGCCGAGCTCACTGGACAGCCCCCCATCCCTGGGCCCCAGCTCAGAGGTGTCTCCCATCCCTACACCGTCGCCTGGGGGCGCTAGCGGTGGCAGCGGCAGCAGCAGCAGCAACGGCCGCTGGAGCAAGGCCTATGATGTGTGCGTGTGCCACAGCGAGGAGGACCTGGTGGCCGCCCAGGAGCTTGTCTCCTACCTGGAGGGCGGCGCCGCCAGCCTGCGCTGCTTCTTGCAGCTTCGCGACGCCACCCCCGGCGGCGCCATCGTGTCCGAGCTGTGCCACGCGCTCAGCAGCAGCCACTGCCGCGTGCTGCTGATTACCCCCGGCTTCCTCCGGGACCCGTGGTGCAGGTACCAGATGCTGCAGGCGCTGAGCGAGGCCCCCGGGGCCGAGGGCCGCACCATCCCCCTGATGTCCGGCCTCAGCAGAGCCGCCTACCCCGCGGAGCTCCGATACATGTACTTCGTGGACGGCCGCGGTCCCGAACGTGGCTTCCGCCAAGTCAAGGACGCTGTCATGCGGTACCTG

IL-18

ATGGCTGCAGAACCAGTAGAAGACAATTGCATCAGCTTTGTGGAAATGAAATTTATTAACAATACACTTTATTTTGTAGCTGAAAATGGCGACCTGGAATCAGATCACTTTGGCAAGCTTGAACCTAAGCTCTCAATCATACGAAATCTGAACGACCAAGTTCTCTTCATTAGCCAGGGAAATCAACCTGTCTTTGAGGATATGCCTGATTCTGACTGTTCAGATAATGCACCCCAGACCATATTTATCATATATATGTATAAGGACAGCCTCACTAGAGGTCTGGCTGTAACCATCTCTGTGCAGTGTAAGAAAATGTCTACTCTCTCCTGTGAGAACAAAATTATTTCCTTTAAGGAAATGAATCCTCCTGATAACATTGATAATGAAGGAAGTGACATCATATTCTTTCAGAGAAGTGTTCCAGGACATGATGATAAGATACAATTTGAGTCTTCATTGTACAAAGGGTACTTTCTAGCTTGTAAAAAAGAGAATGACCTTTTCAAACTCATTTTGAAAAGACAGGATGATAATAGAGATAAATCTGTAATGTTCACTGTTCAAAACCAGAACTAG

AP-1

CACCACCTGCCCCAGCAGATACCCGTGCAGCACCCGCGGCTTCAGGCCCTGAAGGAAGAGCCGCAGACGGTGCCCGAGATGCCCGGGGAAACGCCGCCCCTGTCCCCCATCGACATGGAGTCCCAGGAGCGGATCAAGGCGGAGAGGAAGCGCATGAGGAACCGCATCGCTGCCTCCAAGTGCCGGAAAAGGAAGCTGGAGAGGATCGCGCGGCTGGAGGAAAAAGTGAAAACCTTGAAAGCGCAGAACTCGGAGCTGGCGTCCACTGCCAATATGCTCAGGGAACAGGTGGCCCAGCTTAAACAAAAAGTCATGAACCACGTTAACAGCGGGTGCCAACTCATGCTAACGCAGCAGTTGCAAACGTTTTGA

MAPK1

ATGGCGGCGGCGGCGGCGGCGGGCGCGGGCCCGGAGATGGTCCGCGGGCAGGTGTTCGACGTGGGGCCGCGCTACACCAATCTCTCGTACATCGGCGAGGGCGCCTACGGCATGGTGTGCTCTGCTTACGATAATGTCAACAAAGTCCGAGTCGCCATCAAGAAAATCAGCCCTTTTGAGCACCAGACGTACTGCCAGAGGACGCTGAGAGAGATAAAGATCTTGCTGCGCTTCAGACACGAGAACATCATTGGAATCAACGACATTATTCGGGCGCCGACCATCGAGCAGATGAAAGACGTATACATAGTACAGGACCTCATGGAAACAGACCTCTACAAGCTCTTGAAGACGCAACACCTCAGCAACGACCACATCTGCTACTTTCTCTACCAGATCCTCAGAGGGCTGAAGTATATCCATTCAGCCAACGTGCTGCACCGGGACCTCAAGCCTTCCAACCTGCTGCTCAACACCACCTGCGATCTCAAGATCTGTGACTTTGGCCTGGCCCGTGTTGCAGATCCGGACCACGACCACACAGGGTTCCTGACCGAGTACGTGGCCACGCGCTGGTACCGGGCTCCAGAGATCATGCTGAACTCCAAGGGCTACACCAAGTCCATCGACATTTGGTCCGTGGGCTGCATCCTAGCAGAGATGCTCTCCAACAGGCCCATCTTCCCCGGGAAGCATTACCTCGACCAGCTGAACCACATTCTGGGTATTCTTGGATCTCCGTCGCAGGAAGACCTGAATTGCATAATAAATTTAAAAGCTAGAAACTATCTGCTCTCTCTTCCACACAAAAATAAGGTGCCGTGGAACAGGCTGTTCCCGAATGCTGACTCCAAAGCTCTGGATTTACTGGACAAAATGTTGACGTTCAACCCTCACAAGAGGATCGAGGTGGAGCAGGCTCTGGCCCACCCGTACCTGGAGCAGTACTACGATCCAAGCGACGAGCCCGTCGCCGAAGCACCCTTCAAGTTTGACATGGAATTGGATGACTTGCCCAAGGAAAAGCTCAAAGAACTCATTTTTGAAGAGACTGCTAGATTCCAGCCAGGATACAGATCTTAA

IRAK1

ACCAGCCACCATTGCCATCTCCAGCCCCCTCATCTACCAAGCCCAGCCCGGAGAGCCCCATGCCCCTCCTGCCGGGGGCCCCCTCCTCTTCGTTCTGCTGGCCCCTCCATGAGATTTGCCAGGGCACCCACGACTTCTCAGAGGAGCTCAAGATCGGGGAGGGCGGCTTTGGCTGTGTGTACCGGGCAGTGATGAGGAACACCGTCTATGCTGTGAAGAGGCTCAAGGAGGAGGCTGACCTGGAGTGGACCACAGTGAAGCAGAGCTTCCAGACCGAAGTGCAGCAGCTGTCGCGGTTTCGTCATCCCAACATCGTGGACTTTGCTGGCTACTGTGCTCAGAGTGGCTTCTACTGCCTTGTCTATGGCTTCCTGCCCAATGGCTCCCTGGAAGACCGCCTCCACGTCCAGACACAGGCCTGGCCCCCTCTCTCCTGGCCTCAGCGACTGGACATCCTTCTGGGCACAGCCCGGGCAATTCAGTTCTTACATCAAGATAGCCCCAGCCTCATCCATGGAGATGTCAAGAGTTCCAACGTCCTTCTGGATGAGAGACTGATGCCCAAACTGGGGGACTTCGGCCTGGCCCGTCTCAGCCGGTTTACAGGGGCCAACCCCGGCCAGAGCAGTAGTGTGGCCCGGACTCGGACGGTGCGTGGCACCCTGGCCTACCTGCCTGAGGAGTACGTGAAGACCGGGAGGCTGGCCGTGGACACCGACACCTTCAGCTTTGGCGTGGTGCTGCTGGAGACCCTGGCCGGCCAGAGGGCCGTGAGGATGCATGGCGCCCAGCCCAAGTATCTGAAAGACTTGGTTGAAGAGGAGGCAGAGGAGGCCGGGGTGACCCTGAAGGGCACCCAGACCGCAGTCCAAGGCGGGCCAGCCGCAGACACGTGGGCCGCCCCAGTTGCCGCCCAGATCTACAAGAAGCACCTGGACCCCAGGCCAGGGCCATGCCCACCAGAACTGGGCCTGGCCCTGGGCCAGCTGGCTTGCTGCTGCCTGCACCGCCGGGCCAAGAGGAGACCCCCGATGACCCAGGTGTACCAGACACTGGAGGAGCTGCAGGTGGTGGTGGCAGGGCCATGCCTGGAGCCAGAGGCTGCCAGCCACAGCCCCCCTTCCCCGCAAGAGAACTCCTACGTGTCCACCAGCGGGAGCGCCCTGAGCCGTGCCAGCCCCTGGCAGCCCCTGGTGGTGCCCTCGGGGGCCCAGGCCCAGGCTACAGACTGGCCACACAAAGGTGCCAACCAGCCCGTGGAGAGTGACGAGAGCGTGTCTGACCTCTCTGCTGCCCTGCACTCCTGGCATCTGAGCCCCAGCTGCCCTGCGGGCCCAGGGGCCCCCAGCTGGGTGCCAGCACCCCTCGGGCAGGCTGCCTGCACCCAGGGGGGTGCTGCCCGAGAGTCGAGCTGGGGGAGCGGCCCGGGTCTCCAGCCTACAGCTGTGGAAGGACCCCTCCTGGGCAGTTCCACATCCTCACGGCCCCCGCAGATCGTGATCAATCCGGCGCGACGGAAGATGCTGCAGAAGCTGGCATTGTATGAGGACGGGGTCCTAGACAGCCTGCAGCTGCTCTCGTCCAGCTCTCTCCCAGACTCGGGCCAGGACCTGCGGGACAGGCAGGGGCCCGAAGAGAGAGACGAGTTTCGGAGCTGA

GAPDH

GACCCCTTCATTGACCTTCACTACATGGTCTACATGTTCCAGTATGATTCCACCCATGGCAAGTTCCACGGCACAGTCAAGGCAGAGAACGGGAAGCTCGTCATCAATGGAAAGGCCATCACCATCTTCCAGGAGCGAGATCCTGCCAACATCAAGTGGGGTGATGCTGGTGCTGAGTACGTGGTGGAGTCCACTGGGGTCTTCACTACCATGGAGAAGGCTGGGGCTCACTTGAAGGGTGGCGCCAAGAGGGTCATCATCTCTGCACCTTCTGCTGATGCCCCCATGTTTGTGATGGGCGTGAACCACGAGAAGTATAACAACACCCTCAAGATTGTCAGCAATGCCTCCTGCACCACCAACTGCTTGGCCCCCCTGGCCAAGGTCATCCATGACCACTTTGGCATCGTGGAGGGACTTATGACCACTGTCCACGCCATCACTGCCACCCAGAAGACTGTGGATGGCCCTTCCGGAAAGCTGTGGCGTGATGGCCGAGGGGCTGCCCAGAACATCATCCCTGCTTCTACTGGCGCTGCCAAGGCTGTGGGCAAGGTCATCCCTGAGCTC
